# Amplifying Marginalised Voices Through Advocacy: Community‐Informed Curriculum Development for Cultural Safety in Homeless Health

**DOI:** 10.1111/tct.70314

**Published:** 2025-12-16

**Authors:** Clare Puddifoot, Eleanor Cape, Leanne C Doherty, Ayesha Jawwad, Suzanne Manning, Michelle Doyle, Gary Rutherford

**Affiliations:** ^1^ Ulster University School of Medicine Derry‐Londonderry UK; ^2^ Ulster University Student Success Centre Derry‐Londonderry UK; ^3^ Ulster University School of Health Sciences Derry‐Londonderry UK; ^4^ Western Health and Social Care Trust Derry‐Londonderry UK; ^5^ Addiction Recovery Coaching Derry‐Londonderry UK

**Keywords:** case‐based learning, cultural humility, homelessness health, inclusion health, medical curriculum, patient and public involvement

## Abstract

**Background:**

People experiencing homelessness face significant health inequities, yet their voices remain largely absent from medical education. Partnering with community‐based advocates enables the integration of lived experience and advocacy expertise into curriculum design and delivery.

This study aimed to develop and evaluate a medical education module on homeless health that embeds community‐based advocacy and lived‐experience expertise to advance cultural safety.

**Approach:**

A homelessness health module was collaboratively developed by clinical teachers, researchers, educators and community advocates with lived and professional experience of homelessness. Guided by Kern's Six Steps of Curriculum Development, a targeted needs assessment was conducted through a focus group with community advocates. Insights informed six learning objectives and the design of interactive teaching sessions. The module was delivered to second‐year graduate‐entry medical students and evaluated using open‐ended questionnaires exploring students' understanding of cultural safety and advocacy in homeless health.

**Evaluation:**

Fifty‐three students (33%) submitted open‐ended questionnaires. Deductive content analysis confirmed alignment with themes identified during the needs assessment, with most students recognising life experiences (69%), demographic hardship (65%) and healthcare access barriers (69%) as central to homelessness. Inductive thematic analysis revealed additional themes related to survival priorities and medication adherence barriers.

**Implications:**

Embedding community voices and patient perspectives shaped curriculum content, delivery and evaluation. Students developed compassion, reflexivity and a deeper awareness of inequities. This accessible, scalable model demonstrates how cultural safety can be embedded in inclusion health education.

## 1 Background

Homelessness is a pressing global public health crisis. The United Nations Human Settlements Programme [1] estimates that around 20% of the world's population lives in inadequate housing. In the United Kingdom, one in every 200 households is homeless [2]. Homelessness extends beyond rough sleeping; under the UK Housing Act [3] a person is legally homeless if they lack available accommodation, face violence or domestic abuse, cannot reasonably remain in their current housing, have no legal right of occupation or live in a mobile home or houseboat without a lawful place to reside.

This crisis reflects systemic marginalisation through mechanisms such as poverty and institutional stigma which exclude people from full participation in society [4] and contribute to a disproportionate burden of ill health [5]. Despite greater healthcare needs, people experiencing homelessness encounter significant barriers to accessing health services, including practical challenges and clinician bias [6].

Cultural humility involves critical self‐reflection, recognition of power imbalances and a sustained commitment to addressing health disparities [7, 8]. Cultural safety extends this, calling on health systems and educators to create environments where patients feel empowered by clinical encounters [9]. In health education, this means teaching students how to build respectful relationships with patients, helping them understand how broader social and structural factors affect health and ensuring that institutions take responsibility for creating safe and equitable environments for all.

Collaboration with community organisations and patient advocates, as proposed in the Vancouver Statement [10], provides a tangible means of operationalising cultural safety in health professions education. Such partnerships help reflect the complex realities of lived experience to avoid tokenistic representation, while fostering democratic co‐production and mutual learning [11].

Community voice refers to the inclusion of community members and community organisations in the design and enhancement of curricula. It can be understood as a collective form of patient voice, one that amplifies the lived experiences and broader structural contexts of homelessness ensuring that lived realities are not reduced to individual or exceptionalised narratives [12].

*Community voice refers to the inclusion of community members and community organisations in the design and enhancement of curricula*.

Frameworks such as Hashmi et al. [13] highlight essential competencies of homelessness healthcare education. However, there is little evidence of how to embed these into clinically focused teaching. Homelessness often appears in case‐based learning (CBL) as a peripheral ethical issue, without representation of patients' voices or agency, reinforcing stereotypes rather than challenging them [14]; although service‐learning provides specific opportunities for students to engage with people experiencing homelessness, most initiatives tend to focus on specific demographics of patients and self‐selecting students restricting their reach and impact [15].

This paper describes the design, delivery and evaluation of a homelessness health module for medical students, developed through sustained collaboration with community partners and individuals with lived experience of homelessness.

## 2 Approach

### 2.1 Development Framework

The module was designed by the multidisciplinary Homelessness Health in Medical Education (HHIME) research team at Ulster University, Northern Ireland. Of the five members of HHIME, two had lived experience of homelessness, two were academic clinical educators and three were working with homeless people in the community. All HHIME partners were engaged from the initial needs assessment, design of learning objectives and materials and co‐facilitation of teaching sessions.

**FIGURE 1:**
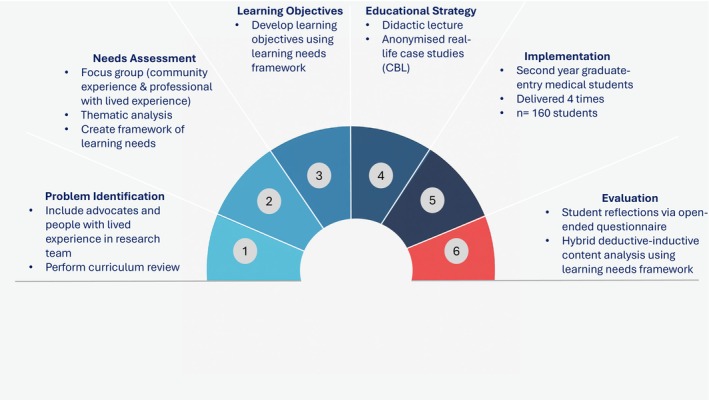
Curriculum development flowchart illustrating the six steps in module development. All members of the HHIME research team were involved throughout.

HHIME's ethos is centred on sustained partnership and power‐sharing, reflecting ethical co‐creation principles [16]. Module development followed Kern's Six‐Step approach to curriculum development (Figure 1) [17], underpinned by constructivist and critically reflective paradigms [18].

Step 1. Problem Identification

The existing curriculum consisted of a single problem‐based learning (PBL) case and two lectures on homelessness, which did not include the patient or advocate voice. This represented a structural omission with potential to reinforce stereotypes and perpetuate exclusion [19].

Step 2. Needs Assessment

To reflect the breadth and diversity of homelessness experiences, we convened a focus group with community experts (*n* = 6) recruited via snowball sampling through HHIME networks. All participants held professional roles in the homelessness sector, many of which involved direct advocacy in healthcare settings. This approach aligns with the concept of community voice as an extension of patient voice, where advocates and frontline professionals articulate collective perspectives and system‐wide barriers that are not captured by individual narratives alone [11, 20].

Two HHIME members participated in the focus group discussion (FGD) to minimise hierarchy, one of whom had lived experience. The FGD was recorded, transcribed verbatim and analysed using reflexive thematic analysis [21] from a constructivist stance. Written consent was obtained and no financial compensation was offered. Five overarching themes were generated through iterative interpretation [21] (Table 1). Themes and categories were used to create a learning needs framework (Table 2).

Step 3. Learning Objectives

**TABLE 1:** Themes generated from needs assessment were aligned to learning objectives and educational approach.

Theme	Integration into module
	Learning objectives (case‐based learning [CBL])
Theme 1. The weight of lived experience	Discuss the complex and varied life experiences of people facing homelessness.
Theme 2. Healthcare systems as obstacles, not supports	Compare barriers to healthcare between people experiencing homelessness and housed populations.
Theme 3. Relational care matters more than clinical skill	Critically discuss how conversations around behaviour change and advice differ in the context of homelessness. Reflect on how unconscious biases towards homelessness and addiction influence communication.
	Learning objectives (Lecture)
Theme 4. Health inequality is embodied	Discuss the health and wellbeing needs of people experiencing homelessness. Discuss health and social care for homeless individuals.
	Educational approaches (Lecture and CBL)
Theme 5. Curriculum as a tool for social change	Use of case based learning and didactic teaching. Inclusion speakers with lived and professional experience. Encouraging reflective practice.

**TABLE 2:** Learning needs framework derived from community focus group themes and categories, integrated with deductive content analysis of student reflections.

Theme	Categories (C)	Illustrative participant quote (community focus group discussion)	Illustrative student quote (post‐module questionnaire)	% Student respondents identifying category
1. The weight of lived experience	C1.1. Life experiences	‘For the young parents that we work with, it would be adverse childhood experiences. They re parents and hardly were parented themselves. There's three generations, maybe, of people coming through, with self‐fulfilling prophecies, and you see the same plans happening to the children.’ (Participant 4)	‘The session highlighted how trauma can play a key role in homelessness especially ACEs [adverse childhood experiences].’ (Student 11) ‘Psychological trauma, adverse childhood events, physical and/or mental health problems, loss of employment, breakdown of relationships.’ (Student 17) ‘I learned more about the difficult circumstances growing up—e.g., poverty, sexual abuse, family members with mental health issues, time spent in foster care.’ (Student 10)	69%
	C1.2. Behaviour as communication	‘Yes they are chaotic, yes there's behaviours that maybe you do not like, unless you have an understanding of how the individual got there, you cannot just make the assumptions straight away 0.9 times out of 10 the individual just wants to be heard or validated and I think we are not that good at doing that.’ (Participant 6) ‘They are treated very different … behaviours dealt with instead of person.’ (Participant 6)	‘To not be scared or feel in danger. The patient is more frightened and scared to seek help than you are.’ (Student 14) ‘Resistance to care plans does not mean unwillingness to access healthcare’ (Student 7)	40%
	C1.3 Demographic hardship	‘In terms of families, I would say, what's happening with the families would be the cost of living and poverty. Where people attend food banks because they just cannot manage … and there's a lot of frustration, loneliness: what they are going to do with their kids, how they are going to clothe their kids, how they are going to keep heating on and food on the table.’ – (Participant 4)	‘Financial situation. Adverse childhood experiences. Addiction.’ (Student 53)	65%
	C1.4 Social isolation:	‘They're in their own tenancy now … completely isolated … no confidence … will not get involved socially.’ (Participant 3)	‘Lack of support system’ (Student 21)	12%
	C1.5 Conflict and violence contribute to homelessness (student‐data generated category)		‘War, conflict’ (Student 32)	5%
2. Healthcare systems as obstacles, not supports	C2.1 Gatekeeper to appointment	‘The barrier that particular some of the [service users] would find would be the receptionists.’ (Participant 5) ‘We have loads of instances where receptionists have basically said, sit down, sit down there someone will see to you, and never take them on.’ (Participant 2)	‘I learned about the problems homeless people have registering with a GP and accessing A&E, e.g., due to long waits and being ignored by staff’ (Student 17)	69%
	C2.2 Literacy and language barriers	‘A lot of them will not go to the doctors, because they feel their language is not on par … they do not understand. [Doctors] are impatient and dismissive.’ (Participant 2) ‘Language barrier would be a big thing for us until they first came.’ (Participant 5)	‘I need to be more aware that it may take longer to build trust with someone who is homeless’. (Student 52) ‘Unable to advocate for themselves. Language barriers.’ (Student 53)	22%
	C2.3 Transport to appointments	‘So you know, you have got lack of money, lack of finances, lack of transport, lack of support. And then you have got physical health is an issue—mobility issues, some with walking aids. And then you have got some taxi drivers who will not take our service users either … and then they are not able to, you know, avail public transport, like buses … so there's a lot of barriers before they can get there.’ (Participant 3) ‘A staff member walked him to the street. Taxi driver drove off. He missed his appointment and was struck off.’ (Participant 3)	‘Cannot come to appointments, travel issues and lack of money’ (Student 4)	16%
	C2.3 Perceived systemic failures (student‐data generated category)		‘Failed by the system’ (Student 22) ‘[lack of] Government support’ (Student 36)	11%
3. Relational care matters more than clinical skill	C3.1 Compassion and personhood	‘There's something about seeing the person and not the problem. But I think we treat people as if they are problematic. When they are repeat attenders or they are coming back again? We start to see that individual as problematic.’ (Participant 6) ‘They feel that they are going to be treated really badly.’ (Participant 2)	‘To treat every patient the same regardless of their situation’ (Student 29) ‘Formulate a tailored plan towards somebody with no fixed abode’ (Student 42)	70%
	C3.2 Better communication by healthcare professionals	‘Communication is huge here. Anybody that I work with, needs to be heard. Now they have difficulty conversing how they feel, so you just have to be patient.’ (Participant 1) ‘Some of them do not make eye contact … as soon as they sense judgement … you have lost them.’ (Participant 5)	‘I am much more aware how my body language can make others feel judged’ (Student 20) ‘That building a foundation of trust is key for patients to keep wanting to pursue healthcare and treatment.’ (Student 14)	29%
	C3.3 Fear of stigma (Student‐data generated category)		‘Fear in general, fear of judgement, potentially encountering shame from care providers. Lack of self‐esteem in believing worth of care’ (Student 31)	24%
4. Health inequality is embodied	C4.1 Dual diagnosis and system bounce‐around	‘If someone has dual diagnosis … they will not be accepted … so they just get pushed from pillar to post.’ (Participant 4)	‘Perhaps increased awareness of the challenges within the healthcare system and lack of joint working as regards homeless patients’ (Student 26)	7%
	C4.2 Health and wellbeing	‘They do not look at the holistic approaches … You're not just an alcoholic or a drug addict. You're a human being.’ (Participant 3) ‘We see mobility issues … lower limb pain … type 2 diabetes … pancreatitis … from long term substance use.’ (Participant 6)	‘Mental health issues and addiction can make it more challenging for some homeless people to engage with the healthcare system and be consistent with treatment plans and appointments.’ ‘False perception that homeless people all suffer addictions’ (Student 22) ‘That homeless stems from much more than just addiction’ (Student 43)	69%
5. Curriculum as a tool for social change	C5.1 Case studies and guest speakers	‘Invite people in to talk bluntly to the students … tell them exactly what we are up against. You know, no frills case studies.’ (Participant 2)	‘I was able to appreciate the difficulties and barriers in accessing care through the case scenario we worked through’ (Student 44)	5%
	C5.2 Experiential learning	‘[Students should] … Come out into the city … Spend some time with these service users.’ (Participant 5) ‘I'm definitely against that because of a couple of comments that our service users used to say, like about being on spectacle here.’ (Participant 2)		0%
	C5.3 Innovation and reflective practice	‘Maybe a unit of innovation … teach people about being kinder … encourage new GPs to look at problems differently.’ (Participant 6) ‘Self‐awareness piece … understand your own [issues] … respond and react without judgement.’ (Participant 6)	‘That well educated people do not become homeless’ (Student 13) ‘That homeless people are dirty’ (Student 5) ‘I had an underlying assumption that certain homeless people were simply not interested in looking after their health’ (Student 11)	87%
6. Reframing non‐compliance (student‐data generated Theme)	C6.1 Hierarchy of priorities (student‐data generated category)		‘They will find it harder to make plans as their lives are more erratic and trickier to balance.’ (Student 47) ‘If they cannot fulfil their own basic needs such as shelter then their health is not a priority to them and they will not seek healthcare.’ (Student 16)	9%
	C6.2 Barriers to medication adherence (student‐data generated category)		‘How easy/difficult it will be for them to adhere to treatment plans needs to be considered’ (Student 52) ‘May not be able to get prescription’ (Student 42) ‘Accessing medication without stigma’ (Student 35)	13%

Themes identified through the needs assessment informed six learning objectives (Table 1), refined through iterative review by focus group participants, HHIME members and independent medical educators.

Step 4. Educational Strategies

A dual‐delivery model was adopted (Table 1). Learning objectives addressing clinical presentations, management and services were delivered in large‐group lectures. Learning objectives addressing aspects of cultural humility and cultural safety were incorporated into two CBL scenarios. Each scenario drew on anonymised patient narratives compiled by the HHIME team, from clinical encounters in homelessness services. Narratives were de‐identified and composited where necessary to prevent deductive disclosure. The CBL scenarios were informed by principles of dialogical, anti‐oppressive learning (Appendix 3).

Step 5. Implementation

The module was delivered four times between 2023 and 2025 to four cohorts of second‐year graduate‐entry medical students (total *n* = 160). Each iteration comprised a lecture delivered to the full cohort (*n* = 40), followed by two CBL sessions conducted in smaller groups (*n* = 20 per group). The first 50‐min case‐based session was facilitated by a general practitioner with experience working with homeless people; the second was facilitated by a homelessness health nurse, and an individual with lived experience of addiction, who participated in a professional teaching role.

Step 6. Evaluation

The module was evaluated through structured student reflections using an open‐ended online questionnaire (Appendix 1) immediately after the session. We used a hybrid deductive–inductive qualitative approach to explore student responses. Summative deductive content analysis was carried out by two independent researchers using the themes and categories generated in the needs assessment as the analytical framework [22] allowing structured comparison between community voice and student reflections. This stage was conducted within a more post‐positivist frame to enable structured comparison between student responses and focus group priorities; the use of two independent coders in this context was to ensure consistent application of the coding framework agreement (Cohen's Kappa = 0.74; 87.4% agreement).

Inductive flexibility was retained to capture additional insights from the student perspective. Researchers independently performed line‐by‐line manual coding of each response and developed inductive themes using comparative analysis, resolving discrepancies by consensus.

### 2.2 Ethics Statement

This study was approved by the Ulster University School of Biomedical Sciences Ethics Committee.

## 3 Evaluation

Fifty‐three students (33%) completed the questionnaire (62% female, 38% male). The majority identified as White (87%) and Irish or Northern Irish (87%), and 30% reported prior personal or professional experience with homelessness, most commonly through healthcare placements, volunteer work with outreach services or brief encounters in personal contexts.

In addition to the themes and categories identified during our needs assessment, one new theme and five new categories were identified through interpretive reflexive analysis. The overall themes are presented in Table 2 with quotes from both the FGD and student reflections.

*There's something about seeing the person and not the problem. … I was able to appreciate the difficulties and barriers in accessing care through the case scenario we worked through* (Student 44)

### 3.1 Theme 1: The Weight of Lived Experience

This theme emphasised that homelessness is driven by lived experiences such as trauma and socioeconomic hardship, shaping both health outcomes and health‐seeking behaviours. Most students identified adverse life experiences (69%) and demographic hardship (65%) as key contributors, with fewer (12%) recognising the impact of social exclusion. A further category, ‘conflict and violence’, emerged from reflexive analysis, with 5% linking unsafe environments such as war, gangs or community violence to health‐seeking behaviour. The community voice also noted that behavioural presentations often overshadow underlying health needs, becoming the primary focus for clinicians. Many students (40%) recognised that negative behaviours may communicate distress rather than ‘non‐compliance’.

*Most students identified adverse life experiences (69%) and demographic hardship (65%) as key contributors*.

### 3.2 Theme 2: Healthcare Systems as Obstacles Rather Than Supports

The community voice described healthcare systems as exclusionary, with administrative gatekeeping, transport to appointments and difficulty understanding language during a consultation identified as barriers. Students most frequently highlighted challenges at the point of access (69%), including difficulties registering with GPs or attending appointments due to transport barriers (16%). Whereas language comprehension was noted by 22%. Reflexive analysis generated a further category of ‘systemic failures’, with 11% describing individuals as being ‘failed by the system’. This recognition reflects a shift towards cultural safety, as students critically acknowledged how institutional practices perpetuate exclusion.

*This recognition reflects a shift towards cultural safety, as students critically acknowledged how institutional practices perpetuate exclusion*.

### 3.3 Theme 3: Relational Care Matters More Than Clinical Skill

This theme highlights that respect, compassion and being valued as a person were often more important than technical skill in supporting engagement with healthcare. The majority of students (70%) reflected on the centrality of compassion, though views diverged: some argued for fairness by treating all patients ‘the same’, while others supported tailored approaches. The community voice emphasised that stigma and biases are often communicated through body language and poor listening skills, while 29% of students reflected on the importance of communication in building trust. Reflexive analysis identified ‘fear of stigma’ as a new category, with 24% acknowledging that patients may anticipate judgement or shame in healthcare settings.

### 3.4 Theme 4: Health Inequality Is Embodied

This theme describes a cycle where unmet needs, stigma and fragmented systems compounded vulnerabilities, leaving people to embody the consequences of inequality. The community voice explained being ‘bounced around’ by services when facing dual diagnosis, illustrating fragmented support. Students recognised similar challenges: 69% noted the impact of dual diagnosis, particularly mental health and addiction, though many also acknowledged that addiction was not universal and that stereotypes risk obscuring wider health and wellbeing needs. A smaller proportion (7%) highlighted how poor joint working within the health system directly affected patient outcomes.

*The community voice explained being ‘bounced around’ by services when facing dual diagnosis, illustrating fragmented support*.

### 3.5 Theme 5: Curriculum as a Tool for Social Change

The community voice suggested using the curriculum as a mechanism to challenge assumptions while innovation and reflective practice were seen as essential. 87% of students acknowledged prior biases, including assumptions such as ‘well‐educated people don't become homeless’, that ‘homeless people are dirty’, or ‘not interested in looking after their health’. These admissions highlight the value of reflective practice in addressing stereotypes.

Case studies and guest speakers were recommended to show students ‘exactly what we're up against’. Experiential learning was also proposed, with calls for students to ‘come out into the city’, though concerns were raised about service users feeling ‘on spectacle’. Only 5% of students mentioned case studies in their reflections, though those who did found them useful.

### 3.6 Theme 6: Reframing Non‐Compliance

Students generated a new theme of ‘non‐compliance’. Several (9%) recognised that survival needs often take precedence over medical care, reframing missed appointments or treatment gaps as reflections of circumstance rather than disinterest. Others described the practical challenges of adherence, such as storing medication without secure housing. These reflections highlight towards the need for trauma‐informed and structurally aware approaches to clinical practice.

## 4 Implications

### 4.1 Embedding Community Voice Strengthens Cultural Safety in Education

Through this module, students recognised the impact of childhood trauma, socioeconomic hardship and structural determinants in driving homelessness. Embedding diverse community voices, via collaboration in curriculum design, shifts learning from individual blame to systemic awareness. Students felt they were able to question their own biases and prejudices while suggesting ways to empower patients by applying this knowledge to adapt their consultation [5, 19]. This shows how community‐informed teaching can drive students towards critical self‐reflection and create an empowering environment, both of which are key aspects of cultural humility and culturally safe practice [9].

### 4.2 Relational Practice Emerges From Community‐Informed Teaching

People experiencing homelessness are often victims of multiple traumatic events such as physical or psychological abuse, neglect, witnessing or experiencing violence [23]. Trauma‐informed care calls for practitioners to focus on trust‐building and promoting sensitivity in patient encounters [24]. Students echoed the community voice in identifying compassion, trust and communication as central to care, while reframing ‘non‐compliance’. Embedding community‐informed teaching promotes trauma‐informed and relational practice, equipping students to engage without judgement.

*Students echoed the community voice in identifying compassion, trust and communication as central to care, while reframing ‘non‐compliance’*.

### 4.3 Community Perspectives Help Students Critique and Challenge Structural Barriers

As a collective modality, the community voice has the potential to extend its reach beyond individual encounters to influence system‐level decision making [12]. In keeping with this, the recognition of systemic failures, gatekeeping and exclusionary practices, illustrates how community‐informed teaching enables students to critique institutions as well as individual encounters. Framing the curriculum as a tool for social change has the potential to prepare future doctors as advocates for equity.

## 5 Limitations

Extending participation to individuals who are currently experiencing homelessness presents significant challenges [25] and would require a co‐designed approach with trusted professionals and peer advocates to facilitate safe recruitment, provide appropriate support and ensure that participation remains ethical, non‐exploitative and trauma‐informed.

While snowball sampling can engage marginalised voices, it risks reinforcing existing networks and gatekeeping. This was addressed by including focus group participants from diverse professional, advocacy and lived experience backgrounds. One participant had a dual role as both focus group member and co‐researcher, introducing potential bias; this was mitigated through group consensus in analysis and transparent documentation of roles.

The use of open‐ended questionnaires may limit the depth of some responses due to time and space constraints. Social desirability bias may also have influenced reflections on sensitive topics such as stigma, in addition to being impacted by only 33% of students participating.

The absence or brevity of commentary in certain areas may itself be meaningful, highlighting areas where further dialogue or alternative learning formats may be required to foster deeper engagement [14, 18].

## 6 Future Application

Sustained impact will depend on longitudinal integration of this content across the curriculum, faculty development to facilitate equity‐focused dialogue and institutional commitment to community partnerships. Future iterations will integrate this module into interprofessional education reflecting its cross‐application and supporting inclusion health across curricula.

*Future iterations will integrate this module into interprofessional education reflecting its cross‐application and supporting inclusion health across curricula*.

## Author Contributions

**Clare Puddifoot:** conceptualization, data curation, formal analysis, investigation, methodology, project administration, resources, supervision, visualization, writing – original draft, writing – review and editing. **Eleanor Cape:** conceptualization data curation, formal analysis, investigation, methodology, writing – review and editing. **Leanne C Doherty:** data curation, methodology, formal analysis, validation, writing – review and editing. **Ayesha Jawwad:** writing – review and editing. **Suzanne Manning:** writing – review and editing. **Michelle Doyle:** conceptualization, formal analysis, investigation, methodology. **Gary Rutherford:** conceptualization, methodology, formal analysis, investigation, writing – review and editing.

## Funding

The authors have nothing to report.

## Ethics Statement

Ethical approval was obtained from the University Biomedical Science Research Ethics Committee.

## Consent

All authors have reviewed the final version of the manuscript and consent to its publication.

## Conflicts of Interest

The authors declare no conflicts of interest.

## Data Availability

The data that support the findings of this study are available on request from the corresponding author. The data are not publicly available due to privacy or ethical restrictions.

## Appendix 1

### Focus Group Semi‐Structure Interview Guide Questions

**Table:**

**Section A: Demographic of service users**
Tell me about the demographic that you represent?
In what ways is this group considered to be marginalised?
**Section B: Experience of health, wellbeing and illness**
Are there specific health and wellbeing issues that are common to the group that your represent?
**Section C: Experience with accessing and receiving healthcare**
What are your own or your service users experience with accessing healthcare?
What aspects of healthcare is successful? And why.
Are there any aspects that are not working? And why.
Can you think of any specific barriers to good healthcare that are related specifically to this demographic?
**Section D: Cultural information relevant to healthcare**
What would you like medical students to be educated on?
What do you think a culturally competent doctor looks like to the people you represent?

## Appendix 2

### Open‐Ended Questionnaire for Students

**Table:**

**(i) Exact Question Prompt**
**Describe:**
• The psychosocial factors that can contribute to someone becoming homeless?
• The difficulties for a person who is homeless, with accessing and receiving care?
• Patient factors in the context of someone experiencing homelessness which may pose a challenge when formulating a management plan? • How would this knowledge change your approach when treating a patient experiencing homelessness in the future?
• The feeling evoked in you when you think about treating people who are homeless? • Your attitude towards a patient who is homeless? • Can you describe an unconscious bias that this session has identified to you?

## Appendix 3

### Case Studies: Anonymised Real‐Life Case Studies Developed in Collaboration With Community Partners for Use in Sessions on Homelessness

**Table:**

**Case 1**
Presentation: 46‐year‐old man livening in temporary accommodation for those facing homelessness attends an outreach sexual health screening at a mobile health unit. He has previously tested positive for Hepatitis C, but has not attended his hospital appointments. Over the following 4 months, a Homeless Public Health Nurse worked closely with him, supporting him through his pre‐treatment assessments, blood tests and the development of a treatment plan, with weekly reviews. Through this partnership, he shared his life story, reflecting openly on his journey from childhood to the present day. Patient's family: Both parents struggled with poor mental health, addiction and domestic violence. His father left the family when he was aged 8. His mother suffered ongoing addiction meant and poverty—no money for food, clothing or heating. Patient's childhood: Age 11, the patient took an early morning milk delivery job to afford a new school uniform—a moment of great personal pride. However, the demands of work affected his performance in school, and he eventually had to give up the job. Shortly after, his mother died due to addiction‐related causes, leaving him without parental care. He entered the care system and lived in a children's home. As a teenager, he was groomed by paramilitary groups who provided him with clothes, phones and a sense of belonging, quickly becoming his new ‘family’. He became involved in rioting and drug dealing, and later witnessed a murder, which had a profound effect on his mental health. He was diagnosed with post‐traumatic stress disorder (PTSD). Patient's adult life: He had married and had three children. Although he initially avoided substance use due to his family history, he began using intravenous drugs in his late 40s as a means of self‐medicating past trauma. His increasing drug use led to the breakdown of his marriage and the loss of access to his children. Recognising the need for change, he attempted to leave the paramilitary group; however, his request was denied and a ‘threat to life’ was issued against him. As a result, he fled the area, went into hiding and ultimately became homeless, isolated from family and support networks. Following medical support and adherence to his treatment plan, his post‐treatment blood results confirmed that the Hepatitis C infection was resolved.
**Case 2**
Presentation: A 59‐year‐old woman from Bolivia attended the homelessness GP clinic with persistent back pain, ongoing for several months. Clinical assessment revealed no red flags. Housing Situation: The patient and her teenage son had been offered a place to sleep on the floor of a kitchen by a ‘good Samaritan’ in the community, after repeated failed attempts to secure rental accommodation. She was not eligible for state support, leaving her and her son extremely vulnerable. Background: Six months earlier, the patient had made the difficult decision to leave her husband and two younger children behind in Bolivia, driven by a desperate need to find work amidst severe political unrest and economic hardship. Arriving in Ireland with hopes of a better future, she has found herself surviving on precarious, cash‐in‐hand jobs, with no social support network to rely on. The emotional burden of separation from her family, combined with chronic financial insecurity, has left her increasingly isolated and vulnerable. Her ability to prioritise her own health—such as attending physiotherapy, maintaining physical activity or managing stress—has been severely compromised. The cumulative impact of housing instability, economic hardship and emotional stress is now taking a visible toll on both her physical and mental well‐being.
